# Chromosome Isolation by Flow Sorting in *Aegilops umbellulata* and *Ae. comosa* and Their Allotetraploid Hybrids *Ae. biuncialis* and *Ae. geniculata*

**DOI:** 10.1371/journal.pone.0027708

**Published:** 2011-11-23

**Authors:** István Molnár, Marie Kubaláková, Hana Šimková, András Cseh, Márta Molnár-Láng, Jaroslav Doležel

**Affiliations:** 1 Agricultural Research Institute of the Hungarian Academy of Sciences, Martonvásár, Hungary; 2 Centre of the Region Haná for Biotechnological and Agricultural Research, Institute of Experimental Botany, Olomouc, Czech Republic; Helmholtz Center Munich, Germany

## Abstract

This study evaluates the potential of flow cytometry for chromosome sorting in two wild diploid wheats *Aegilops umbellulata* and *Ae. comosa* and their natural allotetraploid hybrids *Ae. biuncialis* and *Ae. geniculata*. Flow karyotypes obtained after the analysis of DAPI-stained chromosomes were characterized and content of chromosome peaks was determined. Peaks of chromosome 1U could be discriminated in flow karyotypes of *Ae. umbellulata* and *Ae. biuncialis* and the chromosome could be sorted with purities exceeding 95%. The remaining chromosomes formed composite peaks and could be sorted in groups of two to four. Twenty four wheat SSR markers were tested for their position on chromosomes of *Ae. umbellulata* and *Ae. comosa* using PCR on DNA amplified from flow-sorted chromosomes and genomic DNA of wheat-*Ae. geniculata* addition lines, respectively. Six SSR markers were located on particular *Aegilops* chromosomes using sorted chromosomes, thus confirming the usefulness of this approach for physical mapping. The SSR markers are suitable for marker assisted selection of wheat-*Aegilops* introgression lines. The results obtained in this work provide new opportunities for dissecting genomes of wild relatives of wheat with the aim to assist in alien gene transfer and discovery of novel genes for wheat improvement.

## Introduction

Bread wheat (*Triticum aestivum* L.) is a natural allohexaploid (2n = 6x = 42, AABBDD), which evolved via two rounds of hybridization and polyploidization, involving several species of the *Triticum* and *Aegilops* genera [1]. The gene pool of elite hexaploid wheat is relatively narrow due to thousands of years-long cultivation and domestication [2], undermining the ability to sustain the crop yield and quality under extreme environmental and biotic conditions. The remarkable diversity of related wild species offers a reservoir of novel alleles, favourable genes and gene complexes for wheat breeding by means of interspecific hybridization or transgene technology.

The genus *Aegilops*, which is the most closely related taxon to *Triticum*, contains eleven diploid and twelve polyploid species [3]. Seven distinct genomes (D, S, M, C, U, N and T) were identified in diploids and most of them can be found also in the polyploid *Aegilops* species. Twelve species in the genus contain U and/or M genomes [3] and among them, allopolyploid species evolved from hybridization between diploid *Ae. comosa* Sm. in Sibth. & Sm. (2n = 2x = 14, MM) and *Ae. umbellulata* Zhuk. (2n = 2x = 14, UU). Allotetraploid *Ae. biuncialis* Vis. (2n = 4x = 28, U^b^U^b^M^b^M^b^) and *Ae. geniculata* Roth. (2n = 4x = 28, U^g^U^g^M^g^M^g^) exhibit the largest ecological adaptation ability [3]. These species are valuable sources of useful genes for wheat breeding. Among others, they were used as source of resistance genes to leaf rust, stripe rust, and powdery mildew (*Lr9, Lr57, Sr34, Yr8, Yr40, Pm29*), which were transferred successfully into wheat [4], [5]. Moreover, some genotypes of these species were found tolerant to increased salt levels, drought, frost and heat stresses [6], [7], [8]. Wild relatives of wheat were also studied as potential recipient species for pollen-mediated transgene escape from wheat [9], [10], [11].

Despite extensive research efforts, introgression of favourable agronomic traits from *Aegilops* species to cultivated wheat remains difficult and the production and identification of wheat-*Aegilops* introgression lines by molecular cytogenetic methods is time consuming. Only a small number of U and M genome-specific molecular markers are available [12], [13], [14], a fact that limits marker-assisted selection of wheat-*Aegilops* introgression lines. The lack of suitable markers slows down development of high density genetic and physical maps, mapping of favourable agronomic traits and map-based positional cloning of genes.

Development of molecular markers from particular chromosomes and chromosome arms is an elegant way to saturate genetic maps at regions of interest. Various approaches were employed in several crops to achieve this, such as the use of short-insert chromosome-specific DNA libraries enriched for microsatellites [15], [16], [17] and sequencing ends of clones from chromosome-specific BAC libraries [18], [19]. These approaches relied on construction of libraries from DNA of chromosomes purified by flow cytometry. However, Wenzl et al. [20] demonstrated that DArT markers can be developed in large numbers directly from a small amount of DNA prepared from flow-sorted chromosomes. The advent of the second generation sequencing technology [21] provides an opportunity to skip DNA library construction and identify sequences suitable for development of DNA markers, including SNPs. For example, Berkman et al. [22] sequenced wheat chromosome arm 7DS to 34× coverage using Illumina and assembled low copy and genic regions of this chromosome. The assembly represents approximately 40% of the chromosome arm and all known 7DS genes. Mayer et al. [23], [24] sequenced barley chromosome 1H and arms of chromosomes 2H–7H using 454 to about 2× coverage and assigned 21,766 barley genes to individual chromosome arms and arranged them in a putative linear order based on conserved synteny with genomes of rice, sorghum and *Brachypodium*.

To date, flow-cytometric analysis of mitotic chromosomes has been reported in seventeen plant species, including cultivated cereals with economic importance such as hexaploid and tetraploid wheat, barley, rye, oats, rice and maize [25], [26]. Nevertheless, flow cytometric chromosome analysis and sorting has not been reported in any of their wild relatives. As the technology would greatly aid in transferring genes from wild relatives to cultivated wheat, we set out to explore a possibility of isolating by flow sorting individual chromosomes from *Ae. umbellulata* and *Ae. comosa* and from their natural allotetraploid hybrids *Ae. biuncialis* and *Ae. geniculata*. Chromosomes were sorted from individual peaks of flow karyotypes and were identified by FISH with a set of repetitive DNA probes. DNA amplified from isolated chromosomes was used as a template for PCR with SSR markers with the aim to identify their genomic location in the *Aegilops* species. The results of the present work provide an important step towards analyzing molecular organization of chromosomes in wild relatives of wheat and in developing tools to support alien gene transfer in wheat improvement programmes.

## Materials and Methods

### Plant material

*Aegilops umbellulata* accession MvGB470, *Ae. comosa* accession MvGB1039, *Ae. biuncialis* accession MvGB382 and *Ae. geniculata* accession AE1311/00 were used for flow cytometric chromosome analysis and sorting, for *in situ* hybridization experiments and for microsatellite marker analysis. A partial set of wheat-*Ae. geniculata* disomic addition lines produced by Friebe et al. [27] comprising additions 1U^g^, 2U^g^, 3U^g^, 4U^g^, 5U^g^, 6U^g^, 7U^g^, 1M^g^, 2M^g^, 3M^g^, 5M^g^, 6M^g^, 7M^g^, as well as wheat cv. Chinese Spring and *Ae. geniculata* accession TA2899 (parents of the addition lines) were used to ascertain chromosomal location of wheat SSR markers in the U^g^ and M^g^ genomes.

### Preparation of liquid suspensions of chromosomes

Suspensions of intact mitotic chromosomes were prepared from synchronized root-tips of young seedlings. Cell cycle synchrony was induced after a treatment with hydroxyurea and cycling cells were accumulated in metaphase using amiprohos-methyl [28]. Suspensions of intact chromosomes were prepared according to Vrána et al. [29]. Briefly, 50 roots were cut 1 cm from the root tip, fixed in 2% (v/v) formaldehyde in Tris buffer at 5°C for 20 min. After washing in Tris buffer, the meristem tips were excised and transferred to a tube containing 1 ml of LB01 buffer [30] at pH 9. Metaphase chromosomes were released after homogenization with a Polytron PT1300 homogenizer (Kinematica AG, Littau, Switzerland) at 20,000 rpm for 13 sec. The crude suspension was passed through a 50-µm pore size nylon mesh to remove large cellular debris and stored on ice until analyzed on the same day.

### Flow cytometric chromosome analysis and sorting

The samples were analyzed using a FACSVantage SE flow cytometer (Becton Dickinson, San José, USA) equipped with argon ion laser set to multiline UV and 300 mW output power. Chromosome suspensions were stained with DAPI (4′,6-diamidino-2-phenylindole) at a final concentration of 2 µg/ml and analyzed at rates of 200–400 particles per second. DAPI fluorescence was acquired through a 424/44 band-pass filter. Approximately 30 thousand chromosomes were analyzed from each sample and the results were displayed as histograms of relative fluorescence intensity (flow karyotypes). In order to verify chromosome content of individual peaks on flow karyotypes, one thousand chromosomes were sorted from each peak at rates of approximately 5–10 per second onto a microscope slide into 15 µl drop of PRINS buffer supplemented with 5% sucrose [31], air-dried and used for FISH with probes for DNA repeats that give chromosome-specific fluorescent labeling patterns (see below).

### Preparation of mitotic metaphase spreads

In order to check karyotypes in all *Aegilops* accessions used for flow cytometric analysis, FISH was carried out on mitotic metaphase spreads prepared from root tips as described by Jiang et al. [32].

### Fluorescence in situ hybridization

Genomic DNA was extracted from fresh leaves of *Ae. tauschii* (D genome) and *Secale cereale* (R genome) according to Sharp et al. [33]. Repetitive DNA sequences pSc119.2 and Afa family were amplified from genomic DNA of *S. cereale* and *Ae. tauschii*, respectively, and labelled with biotin-16-dUTP (Roche, Mannheim, Germany) and digoxigenin-11-dUTP (Roche) using PCR as described by Contento et al. [34] and Nagaki et al. [35], respectively. The 18S-5.8S-26S rDNA clone pTa71 [36] was labelled with 50% biotin-11-dUTP and 50% digoxigenin-11-dUTP by nick-translation using standard kits (Roche) following the manufacturer's instructions. Digoxigenin and biotin were detected using anti-digoxigenin-rhodamine Fab fragments (Roche) and streptavidin-FITC (Roche), respectively.

Pretreatment and stringency washes [37] were applied only for the slides containing root tip metaphase cells. These steps were omitted in experiments with flow-sorted chromosomes. Hybridization mix (30 µl per slide), containing 50% formamide, 2×SSC, 10% dextran sulphate, 20 ng of pTa71, and 70 ng each of the pSc119.2 and Afa family probes in the presence of Salmon sperm DNA, was denatured at 80°C for 10 min and stored on ice for 5 min. The chromosome DNA was denatured in the presence of the hybridization mix at 75°C for 6 min and allowed to hybridize overnight at 37°C. For the detection of the hybridization signals, 10 µg/ml each of streptavidin-FITC and anti-digoxigenin-Rhodamin were used. Finally, the slides were counterstained with 2 µg/ml DAPI and examined with Zeiss Axioskop-2 fluorescence microscope using a Plan Neofluar oil objective 63×, N.A. 1.25 (Zeiss, Oberkochen, Germany) equipped with filter sets appropriate for DAPI, FITC and Rhodamin. Images were acquired with a Spot CCD camera (Diagnostic Instruments, Sterling Heights, USA) and compiled with Image Pro Plus software (Media Cybernetics, Silver Spring, USA).

### Amplification of chromosomal DNA

Chromosomes were sorted from each peak on flow karyotype in batches of 25–50,000 (equivalent to 20–40 ng DNA) into PCR tubes with 40 µl sterile deionized water. The chromosomes were treated with proteinase and their DNA was then amplified by multiple displacement amplification (MDA) using Illustra GenomiPhi V2 DNA Amplification Kit (GE Healthcare, Chalfont St. Giles, United Kingdom) as described by Šimková et al. [38]. The amplified DNA was used as a template for PCR with microsatellite markers.

### Microsatellite analysis

Twenty-four microsatellite (SSR) markers derived from bread wheat and wheat A- and D-genome diploid donors were used in this study (Table 1). Genomic DNA was isolated from *T. aestivum* cv. Chinese Spring, *Ae. umbellulata*, *Ae. comosa*, *Ae. biuncialis*, *Ae. geniculata*, and wheat-*Ae. geniculata* addition lines using Quick Gene-Mini80 kit (FujiFilm, Tokyo, Japan) according to the manufacturer's instructions. PCR was carried out in Eppendorf Mastercycler (Eppendorf, Hamburg, Germany) with reaction profiles optimised for each primer pair [39], [40]. PCR reactions were performed in 25 µl reaction volume and the reaction mix consisted of 2× GoTaq Green Master Mix (Promega, Madison, USA) and 0.2 µM primers. 25 ng genomic DNA or 2 ng of MDA-amplified DNA of flow-sorted chromosomes were used as a template. PCR products were separated on 2.5% agarose gel along with the O'RangeRuler™ 50 bp DNA size marker (Fermentas, Vilnius, Lithuania). Electrophoretic patterns were documented and analysed using GeneGenius gel documentation system (Syngene, Cambridge, UK).

**Table 1:** Wheat microsatellite (SSR) markers used in the present study, their assignment to peaks I–IV on flow karyotypes of *Ae. umbellulata*, *Ae. comosa*, *Ae. biuncialis* and *Ae. geniculata* and their positions on *Aegilops* chromosomes.

Marker	Chromosome position in wheat	Assignment to peaks on flow karyotypes				Chromosome position in*Aegilops*	
		*Ae. umbellulata*	*Ae. comosa*	*Ae. biuncialis*	*Ae. geniculata*	Isolated chromosomes#	*Aegilops* additions##
*Xgdm*33	1ABD	I	-	I	I	1U, 1U^b^	na
*Xgwm*164	1A	-	-	-	-	-	-
*Xgwm*232	1D, 5D	-	-	-	-	-	-
*Xgwm*357	1A	-	-	-	-	-	-
*Xcfd*59*	1ABD/6B	I	I	I+III	I+II	1U, 1U^b^	1U^g^
*Xcfd*15	1AD	-	-	-	-	-	-
*Xgwm*391*	3A	III	-	-	-	3U	3U^g^
*Xgwm*383	3DB	-	-	-	-	-	-
*Xgwm*114*	3ABD	-	-	-	II	-	3U^g^
*Xcfa*2134*	3AB	III	-	II	II	3U	3U^g^
*Xgwm*165*	4ABD	IV	I	II+III	III+IV	-	4U^g^
*Xgdm*34*	4D	IV	-	III	III	-	5U^g^
*Xwmc*48*	4ABD	II	-	II	-	6U	na
*Xgwm*160	4A	-	-	-	-	-	-
*Xgwm*205*	5AD	IV	III	III	III	-	5U^g^
*Xgwm*186	6A	-	-	-	-	-	-
*Xgwm*169	6A	-	-	-	-	-	-
*Xgdm*142	7D	-	-	-	-	-	-
*Xgwm*44*	7D	-	III	II	-	-	na
*Xcfa*2040*	7ABD	II	IV	II	II+IV	6U	na
*Xgwm*635*	7AD	-	IV	III	-	-	na
*Xgwm*63	7D	-	-	-	-	-	-
*Xbarc*111	7D	-	-	-	-	-	-
*Xbarc*126*	7D	-	III+IV	-	IV	-	na

*: Markers tested in wheat-*Ae. geniculata* addition lines.

na: *Ae. geniculata* specific fragment was not amplified.

# : Location of the marker on *Aegilops* chromosomes was determined according to the presence in the peaks on flow karyotypes.

## : Location of the marker on *Aegilops* chromosomes was determined according to the presence in the wheat-*Ae. geniculata* addition lines.

*Xgwm*: Röder et al. [39]; *Xgdm*: Pestsova et al. [60]; *Xcfa:* Sourdille et al. [65]; *Xwmc*: Wheat Microsatellite Consortium (http://wheat.pw.usda.gov); *Xbarc*: The US Wheat and Barley Scab Initiative (http://www.scabusa.org).

## Results

### Chromosome analysis using flow cytometry (flow karyotyping)

**Figure 1:**
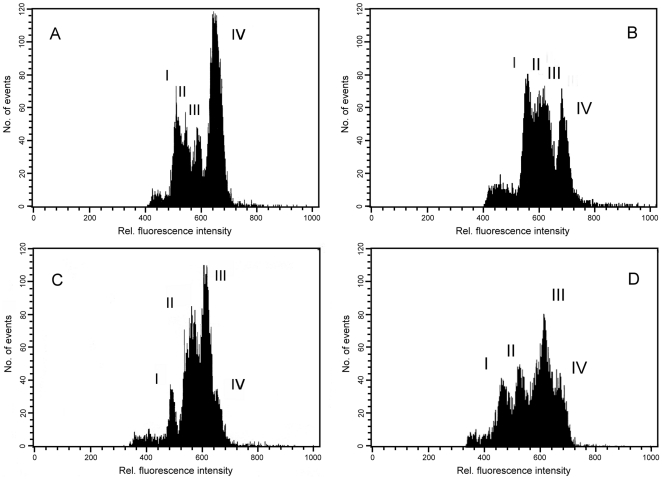
Flow cytometric chromosome analysis. Histograms of relative fluorescence intensity (flow karyotypes) were obtained after the analysis of DAPI-stained chromosome suspensions prepared from *Ae. umbellulata* MvGB470 (2n = 2x = 14, UU) (A); and *Ae. comosa* MvGB1039 (2n = 2x = 14, MM) (B); and from their natural hybrids *Ae. biuncialis* MvGB382 (2n = 4x = 28, U^b^U^b^M^b^M^b^) (C); and *Ae. geniculata* AE1311/00 (2n = 4x = 28, U^g^U^g^M^g^M^g^) (D).

The analysis of DAPI-stained chromosome suspensions prepared from diploid and tetraploid *Aegilops* species resulted in flow karyotypes with four peaks (Figure 1). However, there were differences between the species in the degree of resolution of individual peaks and their position on flow karyotype. While the first three peaks (I–III) on flow karyotype of *Ae. umbellulata* were well discriminated from the composite peak IV (Figure 1A), in *Ae. comosa* peaks II and III could be resolved only partially (Figure 1B). Moreover, chromosome peaks on flow karyotype of *Ae. comosa* were observed at higher fluorescence intensity channels (560–680) as compared to those of *Ae. umbellulata* (channels 480–640). Flow karyotype of allotetraploid *Ae. biuncialis* comprised one single chromosome peak and three composite peaks (Figure 1C). They were observed at similar positions (channels 480–640) as those of *Ae. umbellulata*. Flow karyotype of allotetraploid *Ae. geniculata* comprised four peaks which were located at channels 460–680 (Figure 1D). The range of peak positions in this species corresponded to overlapping ranges of peak positions of *Ae. umbellulata* (channels 480–640) and *Ae. comosa* (channels 560–680).

### FISH on mitotic metaphase spreads

**Figure 2:**
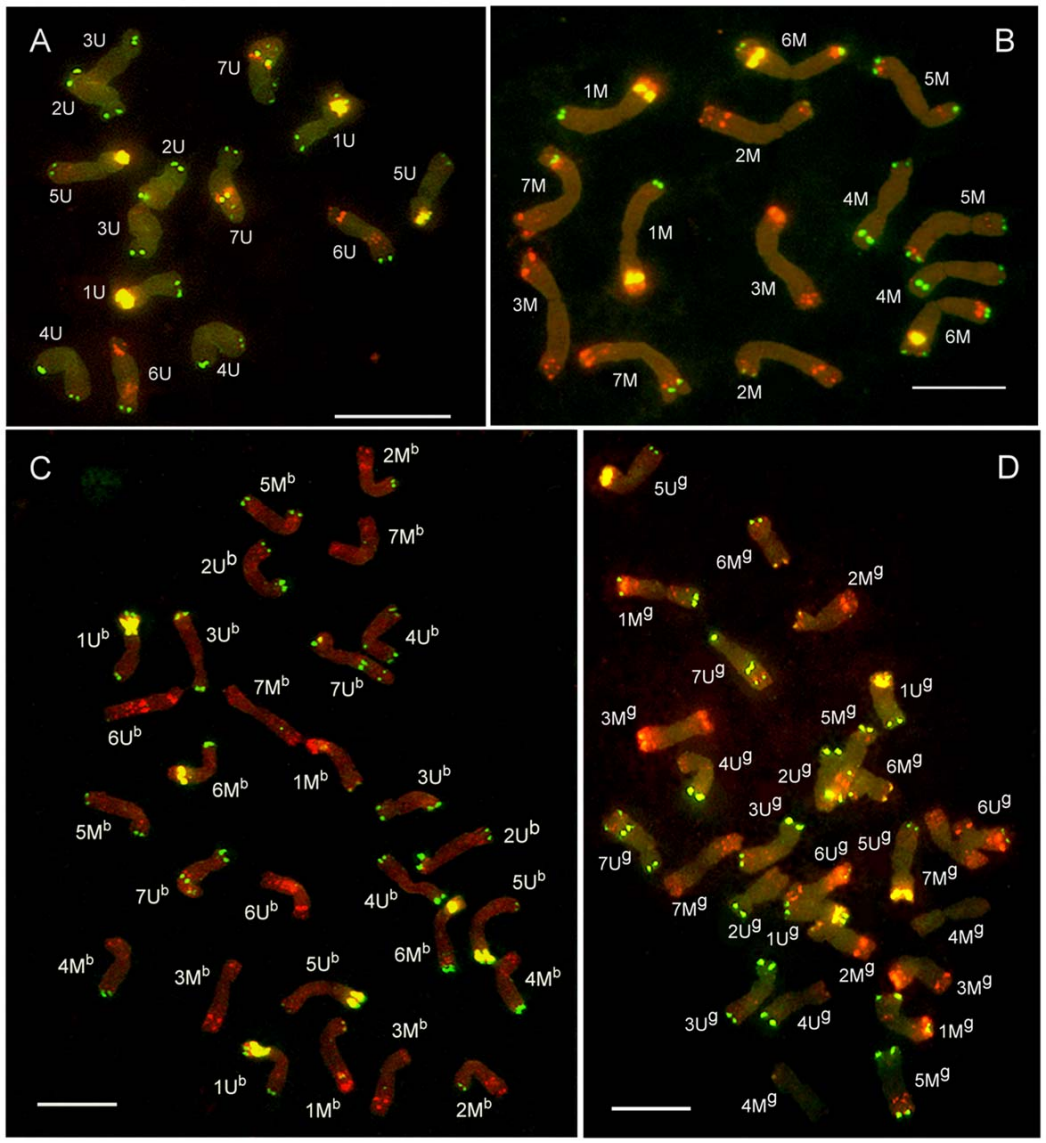
Distribution of DNA repeats on mitotic chromosomes. Fluorescence *in situ* hybridization (FISH) was done with probes for repetitive DNA: pSc119.2 (green), Afa family (red) and pTa71 (yellow) on mitotic metaphase spreads of *Ae. umbellulata* MvGB470 (A); *Ae. comosa* MvGB1039 (B); *Ae. biuncialis* MvGB382 (C); and *Ae. geniculata* AE1311/00 (D). Scale bars = 10 µm.

We could not exclude a possibility that karyotypes of *Aegilops* accessions used in the present work differed from the reference karyotypes [41], [37], [42]. Thus, FISH with repetitive DNA probes (Afa family, pSc119.2, pTa71) was made on mitotic metaphase spreads to obtain chromosome-specific hybridization patterns for *Ae. umbellulata*, *Ae. comosa*, *Ae. biuncialis* and *Ae. geniculata* (Figure 2). Only minor differences were observed in FISH hybridization patterns between the *Aegilops* genotypes used in this study and those used previously [42]. For example, the lack of a terminal pSc119.2 band was observed at the long arm of 2M^b^ and the short arm of 7M^b^ in the *Ae. biuncialis* MvGB382. Chromosome 4M^g^ of *Ae. geniculata* AE1311/00 showed no hybridization signals, while a terminal pSc119.2 signal was detected on the 5U^g^L. However, identification of chromosomes was not hindered by these differences and all chromosomes of diploid and tetraploid *Aegilops* species could be distinguished according to their fluorescence labelling patterns.

### Description of flow karyotypes

**Figure 3:**
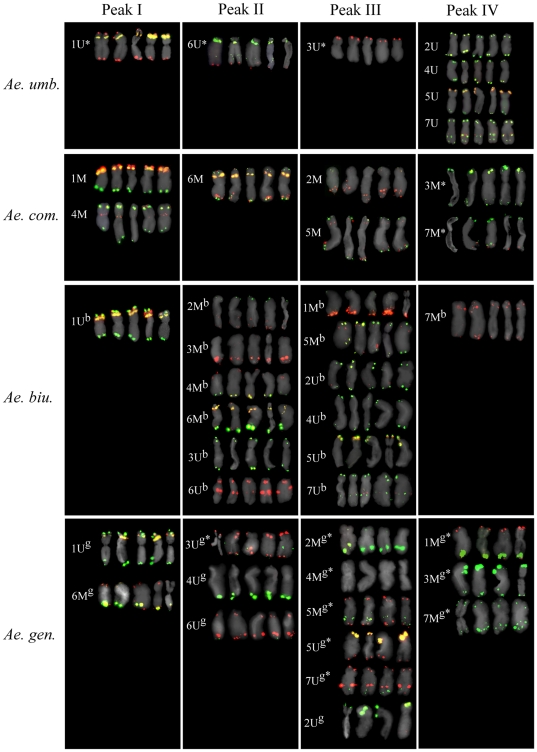
Flow-sorted chromosomes. Mitotic metaphase chromosomes were sorted from individual peaks of flow karyotypes of *Ae. umbellulata*, *Ae. comosa*, *Ae. biuncialis* and *Ae. geniculata*. Sorted chromosomes were identified after FISH with probes for repetitive DNA: pSc119.2 (green), Afa family (red) and pTa71 (yellow) (Inverse labelling of probes is marked by asterisk). The chromosomes are shown in peaks where their frequencies were the highest. For each chromosome, four to five representative examples are given.

Chromosome content of individual peaks was determined after FISH on flow-sorted chromosomes with probes for Afa family, pSc119.2 and pTa71 repeats (Figure 3, Table 2). Strong fluorescent signals of FISH probes were similar to those observed on mitotic metaphase spreads and allowed unambiguous identification of chromosomes in particular peaks of flow karyotypes. In *Ae. umbellulata*, peak I corresponded to chromosome 1 U, which could be sorted at purities >95% (Table 2) Peaks II and III predominantly contained chromosomes 6 U and 3 U, respectively, which were sorted at purities 74.1% and 86.4%, respectively. As expected, the composite peak IV represented chromosomes 2 U, 4 U, 5 U and 7 U. In *Ae. comosa*, peak I contained chromosomes 1 M and 4 M as the main fractions, while the peaks II and III, which were closely spaced, represented mainly chromosomes 6 M and 5 M, respectively. Chromosome 2 M was present in fractions sorted from both composite peaks (II and III). Finally, peak IV contained chromosomes 3 M and 7 M.

**Table 2:** Assignment of chromosomes to peaks of flow karyotypes of *Ae. umbellulata*, *Ae. comosa* and their natural tetraploid hybrids *Ae. biuncialis* and *Ae. geniculata*.

	Genome	Chromosome	Peak I	Peak II	Peak III	Peak IV
*Ae. umbellulata*	U	1	98.9	25.8	4.32	-
		2	-	-	-	31.3
		3	-	-	86.4	-
		4	-	-	-	16.0
		5	-	-	-	30.6
		6	-	74.1	9.25	-
		7	1.03	-	-	22.0
No. of chromosomes analysed			97	120	162	150
*Ae. comosa*	M	1	44.4	16.34	-	-
		2	2.5	30.7	34.2	-
		3	-	-	-	52.4
		4	37.6	-	-	-
		5	3.4	4.28	63.8	-
		6	11.9	47.1	1.8	-
		7	-	-	-	47.5
No. of chromosomes analysed			117	104	108	82
*Ae. biuncialis*	U^b^	1	95.94	0.46	-	-
		2	0.37	1.38	21.35	0.625
		3	1.1	21.75	-	-
		4	-	0.46	7.76	3.125
		5	-	-	12.62	0.625
		6	2.58	8.79	-	-
		7	-	-	15.53	9.375
	M^b^	1	-	-	10.19	1.25
		2	-	25.0	-	-
		3	-	13.8	13.59	-
		4	-	19.44	-	0.625
		5	-	-	18.44	2.5
		6	-	8.79	-	0.625
		7	-	-	0.48	81.25
No. of chromosomes analysed			271	216	206	160
*Ae. geniculata*	U^g^	1	52.0	1.7	-	-
		2	2.6	2.6	24.3	-
		3	1.3	27.8	2.8	-
		4	5.3	28.7	-	-
		5	-	-	21.5	-
		6	0.6	37.4	1.8	-
		7	-	-	11.2	-
	M^g^	1	-	0.8	-	28.8
		2	-	-	12.1	-
		3	-	-	-	42.3
		4	-	-	14.0	3.8
		5	-	-	13.0	-
		6	38.0	0.8	-	-
		7	-	-	-	25.0
No. of chromosomes analysed			150	115	107	104

The numbers represent percentage of particular chromosome type from the whole peak content.

FISH analysis showed significant differences between flow karyotypes of allotetraploids *Ae. biuncialis* (U^b^U^b^M^b^M^b^) and *Ae. geniculata* (U^g^U^g^M^g^M^g^) (Figure 3, Table 2). In *Ae. biuncialis*, chromosome 1U^b^ could be sorted at high purities (>95%) as it was represented by well separated peak I (Figure 1C, Figure 3, Table 2). Peak II contained chromosomes 2M^b^, 3M^b^, 4M^b^, 6M^b^, 3U^b^, and 6U^b^, while peak III represented chromosomes 1M^b^, 3 M^b^, 5M^b^, 2U^b^, 4U^b^, 5U^b^ and 7U^b^. Chromosome 3M^b^ was observed in these two peaks with similar frequency. Peak IV represented largely chromosome 7M^b^, which could be sorted at purities exceeding 80%. On the other hand, peak I of *Ae. geniculata* contained chromosomes 1U^g^ and 6M^g^, while the peak II represented chromosomes 3U^g^, 4U^g^ and 6U^g^. Chromosomes 2M^g^, 4M^g^, 5M^g^, 2U^g^, 5U^g^ and 7U^g^ were found in peak III, while the largest chromosomes 1M^g^, 3M^g^ and 7M^g^ were represented by peak IV (Figure 1D, Figure 3, Table 2).

### Assignment of microsatellite markers to peaks on flow karyotypes

**Figure 4:**
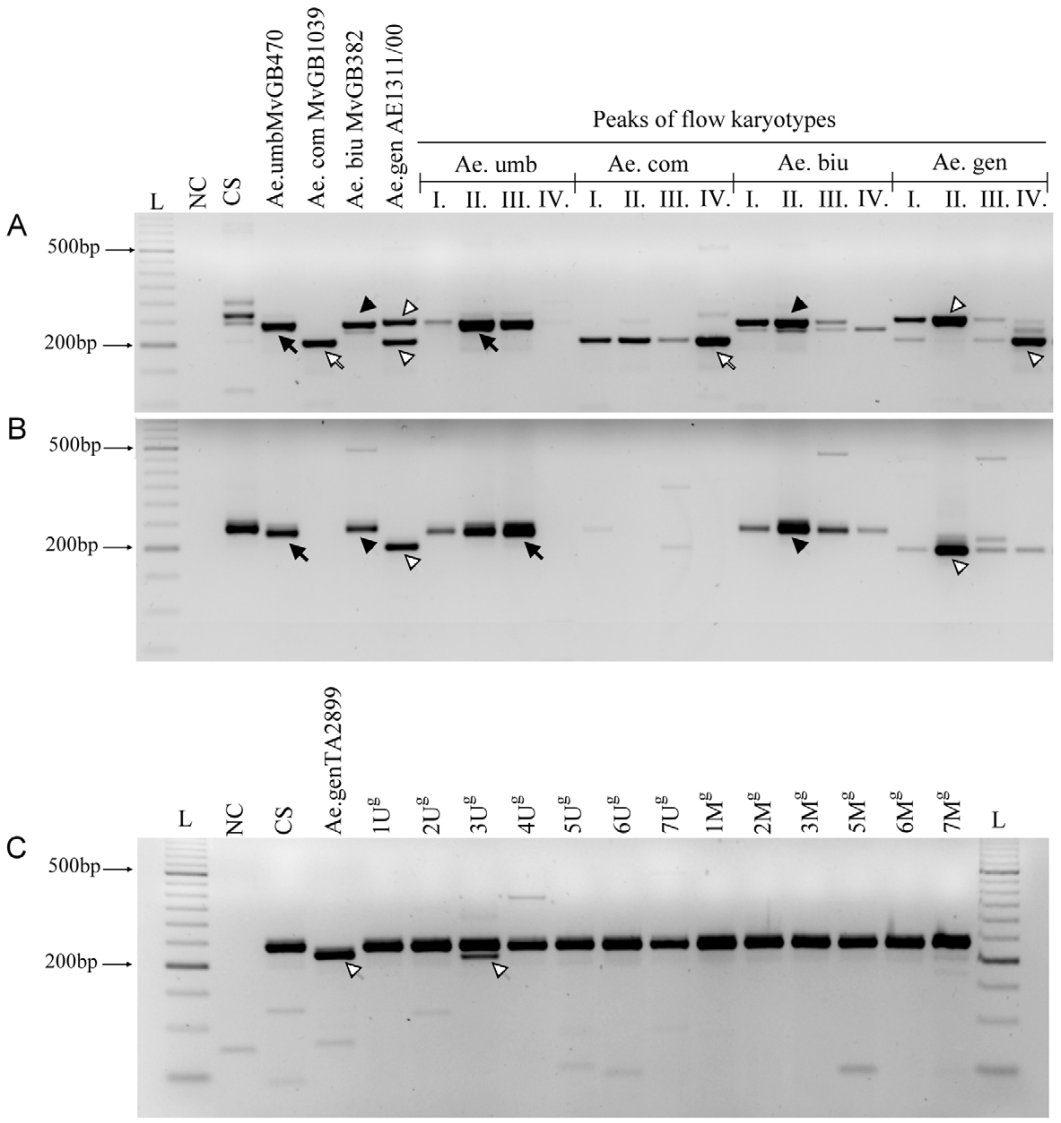
Physical mapping of microsatellite (SSR) markers. PCR products of wheat SSR markers *Xcfa2040* (A); and *Xcfa2134* (B–C) amplified from wheat cv. Chinese Spring, *Ae. umbellulata* MvGB470, *Ae. comosa* MvGB1039, *Ae. biuncialis* MvGB382 and *Ae. geniculata* AE1311/00 and from chromosomes of particular peaks of flow karyotypes (I, II, III, IV) (A–B); and from wheat-*Ae. geniculata* TA2899 addition lines (C). Black arrows, white arrows, black arrowheads and white arrowheads indicate bands specific for *Ae. umbellulata*, *Ae. comosa*, *Ae. biuncialis* and *Ae. geniculata*, respectively.

A set of wheat SSR markers was mapped to *Aegilops* chromosomes using genomic DNA and subgenomic DNA from individual peaks of flow karyotypes. Out of twenty-four SSR markers, thirteen showed one or two species-specific PCR products in at least one of the *Aegilops* species (Figure 4, Table 1). PCR products of these markers were also detected with the expected size in one or more peaks of the given species (Figure 4). For example, marker *Xcfa2040*, which is specific for wheat chromosome group 7 (7ABD), gave a single PCR product in diploid *Ae. umbellulata* and *Ae. comosa* and in allotetraploid *Ae. biuncialis*, while two products of different size were observed in allotetraploid *Ae. geniculata* (Figure 4A). PCR on chromosomes sorted from peaks I, II and III yielded different amounts of the product with size typical of *Ae. umbellulata*. The strongest PCR product was detected at peak II, which comprises mainly chromosome 6U (Table 2). The marker gave the strongest PCR product on chromosomes isolated from peak IV of *Ae. comosa*, which contains chromosomes 3M and 7M. The strongest product of *Xcfa2040* was observed in *Ae. biuncialis* in the 6U^b^ rich peak II, while in *Ae. geniculata* one of the bands relating to *Xcfa2040* was produced predominantly from the 6U^g^ rich peak II, whereas the other *Xcfa2040* band was produced from peak IV comprising 3M^g^ and 7M^g^. The positions of the strongest PCR products indicate that the *Xcfa2040* marker has a locus on 6U chromosomes of *Ae. umbellulata*, *Ae. biuncialis* and *Ae. geniculata* and another locus on chromosome 3M and/or 7M in *Ae. comosa* and *Ae. geniculata*.

Similar to *Xcfa2040*, most of the markers gave specific products for more than one peak, which was consistent with the cytological results (Table 2) and could be explained by overlap of the peaks in the flow karyotype. Other explanation might be that SSR markers, which are multilocus markers with high variability, may have different loci on chromosomes represented by different peaks. Therefore, twelve out of the thirteen markers giving PCR products in the *Aegilops* species were tested in a set of wheat-*Ae. geniculata* addition lines in order to support their chromosomal assignment based on flow-sorted chromosomes. For example, a single band of the marker *Xcfa2134* was found strongest in peaks containing most of the 3U chromosomes (peak III in *Ae. umbellulata* and peak II in *Ae. biuncialis* and *Ae. geniculata*) (Figure 4B). This marker gave *Aegilops*-specific PCR product only in the 3U^g^ addition line (Figure 4C) confirming that this marker has locus only on the 3U chromosomes. The weaker bands observed in the neighbouring peaks most probably resulted from the overlap of the peaks. Thus, the markers were assigned to peaks showing the most intense PCR products. Table 1 provides a summary of assignments of SSR markers to particular peaks of flow karyotypes and their deduced chromosomal locations.

Because of genetic diversity between *Ae. geniculata* AE1311/00 used for chromosome sorting and the parental genotype TA2899 of the addition lines, only seven SSR markers gave PCR products on *Ae. geniculata* TA2899 whose chromosomal location could be identified on the wheat-*Ae. geniculata* addition lines thanks to fragment length polymorphism between *T. aestivum* and *Ae. geniculata* (Table 1). The chromosomal positions of markers agreed well with the PCR results of the peak-specific DNA and with the cytological data (Table 2).

In summary, out of the twenty-four SSR markers tested, ten (41.6%) could be assigned to individual chromosomes of *Ae. umbellulata* (1U: *Xgdm33*, *Xcfd59*; 3U: *Xgwm391*, *Xcfa2134*; 6U: *Xwmc48*, *Xcfa2040*), *Ae. biuncialis* (1U^b^: *Xgdm33*, *Xcfd59*) and *Ae. geniculata* (1U^g^: *Xcfd59*; 3U^g^: *Xgwm391*, *Xgwm114*, *Xcfa2134*; 4U^g^: *Xgwm165*; 5U^g^: *Xgdm34*, *Xgwm205*). More than one locus was identified for five of them. Possibly more loci exist also for other markers, whose chromosomal location could not be identified unambiguously as they were located in composite peaks, but the *Aegilops*-specific bands were not detected on the addition lines.

## Discussion

Chromosome analysis and sorting (flow cytogenetics) has been developed in species belonging to tribe *Triticeae* with high socio-economic importance such as bread wheat, durum wheat, barley and rye [29], [28], [43], [44]. The technology has been a foundation of chromosome genomics [26], an elegant approach to tackle the complex genomes of these crops. Chromosome genomics facilitates the analysis of molecular structure of chromosomes and chromosome arms, high-throughput development of markers, construction of ready-to-sequence physical maps and positional gene cloning. The present work extends the potential of chromosome genomics to wild genetic resources of wheat by establishing flow cytogenetics in two allotetraploid species *Ae. biuncialis* and *Ae. geniculata* and their diploid progenitors *Ae. umbellulata* and *Ae. comosa*. The protocol for preparation of suspensions of intact chromosomes and chromosome sorting is based on protocols developed for wheat [28], [29] and our results in *Aegilops* species confirm that the original protocol for preparation of suspensions of intact mitotic chromosomes from synchronized root tips [45] can be modified to a range of species.

Flow cytometric analysis of chromosome fluorescence intensity results in a distribution of relative fluorescence intensity (flow karyotype), which is typical for given species and genotype [46]. However, it is rare that every chromosome is represented by a single peak on flow karyotype and it is critical to characterize a flow karyotype and determine chromosome content of each peak. One of the most efficient methods to assign chromosomes to the peaks is FISH [28]. In this work we employed FISH with probes for pSc119.2, Afa family and pTa71 repeats, whose genomic distribution was described previously in *Ae. biuncialis* and *Ae. geniculata* as well as in their diploid progenitors *Ae. comosa* and *Ae. umbellulata* [37], [42], [47].

The fact that chromosome peaks on flow karyotype of *Ae. comosa* were shifted towards higher fluorescence channels as compared to *Ae. umbellulata* suggests that the average size of the M genome chromosomes, and hence the size of the M genome is larger than that of the U genome. These results agree with published 1C values of *Ae. umbellulata* (5.05 pg DNA) and *Ae. comosa* (6.18 pg DNA) [48] and indicate that their genomes underwent different evolutionary changes. Both allotetraploids originated from natural hybridization between *Ae. umbellulata* and *Ae. comosa* and have the same genomic constitution (UUMM). The 1C value of *Ae. biuncialis* (11.3 pg) is close to the sum of *Ae. umbellulata* and *Ae. comosa* genome sizes (11.23 pg DNA) [48], while that of *Ae. geniculata* (10.29 pg DNA) is significantly less [49]. The evolution of polyploid species is often accompanied by genome rearrangements, including elimination of certain DNA sequences [50], [51]. Thus, the 1C values suggest that *Ae. biuncialis* originated more recently from its diploid ancestors than *Ae. geniculata*. This hypothesis is in good agreement with a genetic diversity study in which *Ae. biuncialis* showed a much closer relationship to both of its ancestors than *Ae. geniculata* [13]. These evolutionary changes seem to be manifested at chromosome level as significant differences were observed in chromosome content of peaks on flow karyotypes between two tetraploid *Aegilops* species, reflecting differences in relative size of homoeologous chromosomes.

In fact, six chromosomes (4U, 1M, 2M, 3M, 4M and 6M) showed different peak locations in flow karyotypes of *Ae. biuncialis* and *Ae. geniculata*. Interestingly, with the exception of chromosome 3M, substantial differences were observed for the same chromosomes in the distribution of satellite sequences and microsatellite motifs GAA_n_ and ACG_n_ [42]. Moreover, during meiosis in F_1_ hybrids between wheat-*Ae. geniculata* addition lines and *Ae. biuncialis*, ring bivalents between *Ae. geniculata* and *Ae. biuncialis* chromosomes were not observed in those F_1_ hybrids, which involved chromosome additions 4U^g^, 1M^g^, 3M^g^, 4M^g^ and 6M^g^ [27]. It may be hypothesized that differences in repetitive DNA and size of these chromosomes may account for blocking the chromosome pairing, which may in turn lead to reproductive isolation of *Ae. biuncialis* and *Ae. geniculata*. Different location of chromosomes 4U, 1M, 2M, 3M, 4M and 6M on flow karyotypes is also in good agreement with the previous results based on STS, RFLP and AFLP markers and karyotype analysis [37], [47], [52], [53], [54], indicating that U-genome chromosomes exhibit less interspecific variability than the M genome partners in the *Aegilops* species.

Analysis of sorted chromosome fractions by FISH showed that chromosome 1U could be sorted at more than 95% purity from *Ae. umbellulata* as well as from *Ae. biuncialis*. However, relatively pure (74–86%) fractions of chromosomes 6U, 3U and 7M^b^ could also be obtained from these species. Further improvements in the protocol might lead to increased purity of these chromosome fractions in the future. Other chromosomes of the *Aegilops* species formed composite peaks on flow karyotypes and could be sorted as groups of two to four. A possibility to purify by flow sorting chromosome 1U from allotetraploid *Ae. biuncialis* and from its U-genome diploid progenitor *Ae. umbellulata* provides opportunities for chromosome survey sequencing using next generation technology. This is a cost effective approach to study molecular composition of individual chromosomes, identify and assemble most of the low-copy sequences and genes on them and order the genes on chromosomes [22], [23], [55]. The homoeologous group 1 chromosomes of the *Triticeae* harbour many important genes such as seed storage proteins (HMW glutenins) determining the bread-making quality in wheat and genes conferring resistance to fungal pathogens (leaf rust, stem rust, yellow rust) and pests (green bug) [56], [57]. Until now, only one study focused on targeted development of U and M genome-specific molecular markers using S-SAP (Sequence-Specific Amplified Polymorphism) technology [13]. However, next-generation survey sequencing of 1U may provide almost unlimited numbers of sequences suitable for development of markers, including SSR, ISBP, and SNP.

The homoeologous group 1 chromosomes 1H and 1R can be sorted from barley and rye, respectively [43], [44], short arm of 1R (1RS) can be sorted from wheat-rye ditelosomic addition line [58] and short and long chromosome arms of wheat homoeologous group 1 (1ABD) can be sorted from telosomic lines of wheat [46]. Chromosome 1H of barley and chromosome arms of homoeologous group 1 of wheat were survey sequenced recently [23], [55]. Thus, comparative sequence analysis of chromosome 1U from *Aegilops* species with the group 1 chromosomes of wheat, barley and rye is now feasible and may promote the discovery of new wild alleles of important genes for wheat breeding. Such study would also provide detailed insights into the structural changes, which accompanied the evolution of *Triticeae* genomes and the homoeologous group 1, in particular. Other important use of flow-sorted chromosomes has been the construction of BAC libraries [19], which facilitate targeted development of markers, positional gene cloning and construction of sequence-ready physical maps [17], [18], [59]. To date, BAC libraries have been constructed from 17 chromosomes of wheat (http://lmcc.ieb.cz/dna-libraries). The advance reported in the present work opens a way for constructing BAC libraries from chromosomes 1U of *Aegilops*.

Our results obtained after PCR with wheat SSR markers on DNA amplified from flow-sorted *Aegilops* chromosomes confirm previous observation on suitability of MDA-amplified chromosomal DNA for molecular marker analysis [38]. On the other hand, our results also indicate that the flow-sorted chromosomes can be applied for physical mapping of molecular markers, especially when cytogenetic stocks representing the whole chromosome complements are not available. It must be noted however, that in some cases PCR products of SSR markers specific for the longer chromosomes were also detected in the fraction of shorter chromosomes (peaks I and II), albeit with low intensity. The contamination could be due to fragments of longer chromosomes and their chromatids. Further improvement of the protocol may increase the purity in sorted chromosome fractions.

Wheat SSR markers have been used widely for molecular characterization of different *Aegilops* species including *Ae. biuncialis* and *Ae. geniculata* [12], [14], [60], [61], [62]. However, the present as well as the earlier observations [14], [63] suggest a limited efficiency of wheat SSR markers for characterization of U- and M-genome chromosomes. In this work, *Aegilops* specific loci were identified for ten wheat SSR markers, four of them (*Xgwm114*, *Xgwm165*, *Xgdm34* and *Xgwm205*) were mapped earlier on the same *Aegilops* chromosomes [62]. The markers which identify loci on *Aegilops* genomes are suitable for marker assisted selection of wheat-*Aegilops* chromosome addition and translocation lines.

We have found that most of SSR markers were located on the same homoeologous group chromosomes in the *Aegilops* species as in wheat. However, three markers located in different homoeologous goups. Markers *Xgdm*34 and *Xwmc*48 specific for the group 4 chromosomes of wheat were located on *Ae. umbellulata* chromosomes 5U and 6U, respectively, while the marker *Xcfa*2040 specific for the homoeologous group 7 of wheat was identified on chromosome 6U of *Ae. umbellulata*. These results are consistent with the study of Zhang et al. [64] who mapped wheat RFLP markers on *Ae. umbellulata* chromosomes and showed that at least eleven rearrangements differentiate the D genome of wheat from that of *Ae. umbellulata*. For example, SSR marker *Xcfa*2040, which is located on chromosome 6U according to the present study was physically mapped on long arm of wheat chromosome 7D, bin 7DL3-0.82-1.00 [65]. The RFLP marker *XksuD*2 was located at similar bin position [66]. The *XksuD*2 marker was identified later on long arm of chromosome 6U [64], indicating that the wheat chromosome region represented by markers *Xcfa*2040 and *Xksu*D2 changed its chromosomal location in *Ae. umbellulata* due to the chromosomal rearrangements after the evolutionary divergence of wheat and *Ae. umbellulata*.

The regions represented by SSR marker *Xwmc*48 and linked RFLP markers *Xpsr*144 and *Xpsr*139 have also changed their chromosomal location in *Ae. umbellulata* relative to wheat as these markers were mapped on chromosomes 6U and 4D (bins 4DS1-0.57-0.67 and 4DS3-0.67–0.82), respectively [64], [65], [67]. Similar genome modification was indicated by the group 4-specific SSR marker *Xgdm*34 and a linked RFLP marker *Xpsr*164 [65], [67], which were mapped to chromosome 5U in the present study and by Zhang et al. [64].

To conclude, this study marks an important step forward in development of flow cytogenetics for wild genetic resources of wheat. The flow karyotypes of *Ae. umbellulata* and *Ae. comosa* and their natural hybrids *Ae. biuncialis* and *Ae. geniculata* were characterized and chromosome content of all peaks on karyotypes was determined for the first time. While only chromosome 1U could be sorted from *Ae. umbellulata* and *Ae. biuncialis* with standard karyotype, the results indicate a possibility to sort other chromosome types using accessions with rearranged karyotypes [42]. The ability to purify chromosomes in *Aegilops* species opens avenues for rapid physical mapping DNA sequences to particular chromosomes using PCR and DNA arrays, construction of chromosome-specific BAC libraries and next generation sequencing to identify low-copy and genic sequences and develop new markers. Wheat SSR markers which were assigned to *Aegilops* chromosomes in this study could be used in pre-breeding programmes to select chromosome segments carrying agronomically useful genes in wheat-*Aegilops* translocation lines.
