# Correction: Chromosome Isolation by Flow Sorting in *Aegilops umbellulata* and *Ae. comosa* and Their Allotetraploid Hybrids *Ae. biuncialis* and *Ae. geniculata*

**DOI:** 10.1371/annotation/10931d7b-a866-4628-8c84-8c299c972080

**Published:** 2011-12-27

**Authors:** István Molnár, Marie Kubaláková, Hana Šimková, András Cseh, Márta Molnár-Láng, Jaroslav Doležel

There was an error in the funding statement. The correct funding statement is: "This work was supported by the Hungarian National Research Fund (PD83444), by the János Bolyai Research Scholarship of the Hungarian Academy of Sciences (for MI and CSA), the Czech Ministry of Education, Youth and Sports (grant awards LC06004 and OC08025) and the European Regional Development Fund (Operational Programme Research and Development for Innovations No. ED0007/01/01). The funders had no role in study design, data collection and analysis, decision to publish, or preparation of the manuscript.”

